# Hurricane Activity and the Large-Scale Pattern of Spread of an Invasive Plant Species

**DOI:** 10.1371/journal.pone.0098478

**Published:** 2014-05-30

**Authors:** Ganesh P. Bhattarai, James T. Cronin

**Affiliations:** Department of Biological Sciences, Louisiana State University, Baton Rouge, Louisiana, United States of America; University of New South Wales, Australia

## Abstract

Disturbances are a primary facilitator of the growth and spread of invasive species. However, the effects of large-scale disturbances, such as hurricanes and tropical storms, on the broad geographic patterns of invasive species growth and spread have not been investigated. We used historical aerial imagery to determine the growth rate of invasive *Phragmites australis* patches in wetlands along the Atlantic and Gulf Coasts of the United States. These were relatively undisturbed wetlands where *P. australis* had room for unrestricted growth. Over the past several decades, invasive *P. australis* stands expanded in size by 6–35% per year. Based on tropical storm and hurricane activity over that same time period, we found that the frequency of hurricane-force winds explained 81% of the variation in *P. australis* growth over this broad geographic range. The expansion of *P. australis* stands was strongly and positively correlated with hurricane frequency. In light of the many climatic models that predict an increase in the frequency and intensity of hurricanes over the next century, these results suggest a strong link between climate change and species invasion and a challenging future ahead for the management of invasive species.

## Introduction

Biological invaders are widespread and increasing in number in marine, freshwater and terrestrial ecosystems worldwide [1], [2], and because their occurrence is often linked to climate change, the rise in invasions is expected to continue into the future [3]–[7]. Moreover, successful invaders can have dire consequences for the persistence of native species, food-web structure, ecosystem functioning [8], [9], and, ultimately, the economy [10]. Mechanisms promoting establishment and spread of invasive species in particular habitats (local scale) have been well studied and include possession of traits that facilitate establishment and invasion (e.g., strong dispersal ability, high reproductive rate, superior competitive ability) and release from natural enemies [11], [12].

Alterations of habitat characteristics by natural and anthropogenic disturbances, or change in disturbance regimes, are quite often associated with invasion success [13]–[15]. Disturbances benefit invasive species by reducing competition with resident species and increasing resource availability [13], [16]. Large scale disturbance events such as hurricanes, cyclones and typhoons have long been associated with the establishment and spread of invasive species [17], [18]. However, to date, few studies have considered whether the history of such extreme disturbance events has influenced invasion success at local scales [7], [16], and no studies have addressed whether these types of disturbances affect the patterns of establishment and spread at regional or continental spatial scales. For example, in the northern hemisphere historical patterns of spread of invasive species may be greater in the south where hurricanes are more frequent and intense than in the north. As such, range expansion and spread of an invader may be driven by disturbance regimes. The relevance of studying hurricane effects on the establishment and spread of invasive species is magnified by the expectation that hurricane activity, particularly high-intensity hurricanes, may increase with global climate change [19]–[21].

We studied the effect of storm and hurricane activities on the growth of patches of common reed, *Phragmites australis*, in the coastal wetlands of the eastern United States of America. Indigenous and/or introduced haplotypes (based on a microsatellite analysis of chloroplast DNA) of *P. australis* are found on all continents except for Antarctica, and in some cases the introduced haplotypes are recognized as aggressive invaders [22], [23]. Historically, *P. australis* has been an uncommon species of the wetlands of North America for millennia [24]. In the past 150 years, an introduced Eurasian haplotype has spread rapidly in both coastal and inland marsh ecosystems of North America, particularly near the Atlantic Coast [22]. An additional haplotype that most likely originated in Africa and is present in all of the Gulf Coast states (Gulf-Coast haplotype) [25] is also spreading locally and expanding its range to the southwestern US [26], [27]. It is unclear whether this haplotype’s appearance into the Gulf Coast region was facilitated by human activities or the result of a natural range expansion from Central and South America. Other non-native haplotypes of *P. australis* are present in North America, but they appear to have very restricted distributions (particularly, within the Mississippi River Delta) [25]. Marshes that have been invaded by *P. australis* have been characterized by the loss of native plant species, reduced diversity and altered composition of associated faunal communities, and changed ecosystem processes such as nutrient cycling and hydrological regimes [28]–[30].

**Figure 1:**
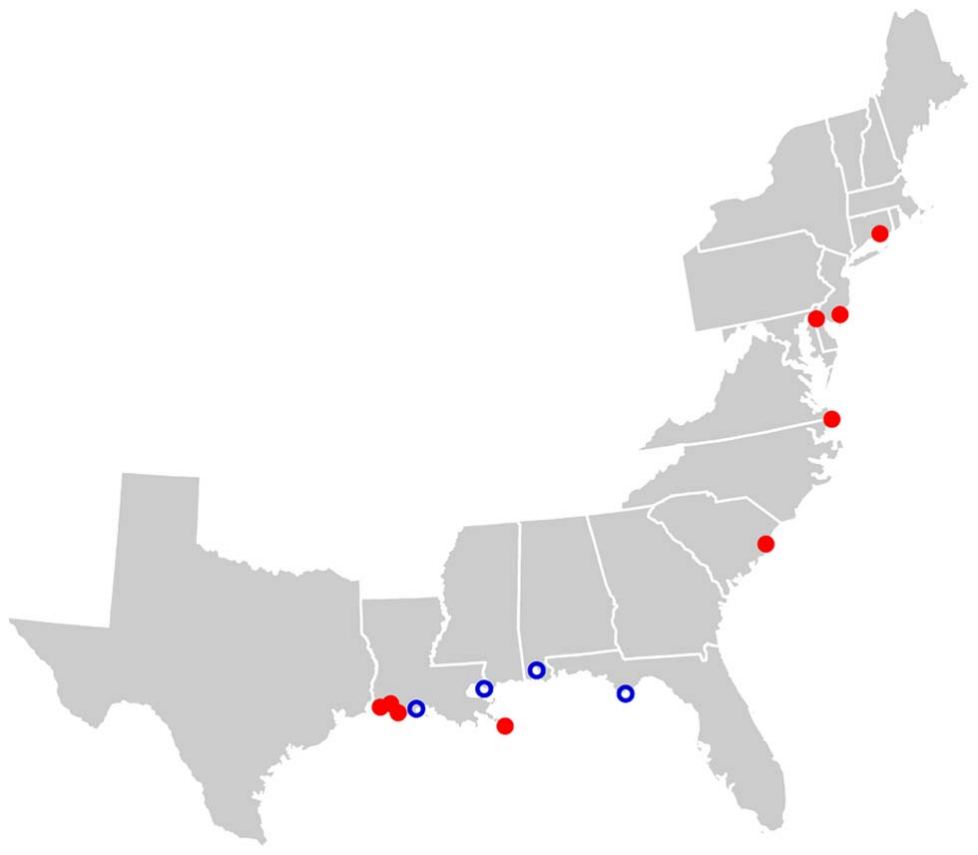
Location of study sites along the Gulf and Atlantic Coasts of the United States. Filled and open symbols represent sites occupied by Eurasian and Gulf-Coast haplotypes of *P. australis* respectively.

In spite of the serious ecological and economic impacts of *P. australis* invasion, almost nothing is known about the factors responsible for the continent-scale patterns of spread of these invasive haplotypes in North America. Using historical aerial images (spanning 5–27 years), we determined the growth rate of *P. australis* patches within each of 13 marsh sites (9 inhabited by the Eurasian and 4 inhabited by the Gulf-Coast haplotype) distributed along the Gulf and Atlantic Coasts of the US (Figure 1, Table 1). For each site, we estimated wind speeds of all storms, counting only those that qualified as a tropical or subtropical storm (maximum sustained wind speeds of 65–119 km/h) or hurricane (≥119 km/h). By dividing storms into these two wind-speed categories, we were able to test the *a priori* prediction that growth rates of *P. australis* patches were more strongly related to the frequency of more intense storms.

**Table 1:** Description of the study sites and duration of the study.

Site	State	Longitude	Latitude	Area(km^2^)	Period ofimagery	Imagerydates	Haplotype	Numberofpatches	Initial patcharea (m^2^)(mean ± SE)	Number of	
										Tropicalstorms	Hurricanes
Pettipaug Yacht Club	CT	−72.38	41.37	2.21	1991–2010	1991, 1994, 1997, 2005,2008, 2010	Eurasian	12	3686.20±2328.97	3	1
Estell Manor Park	NJ	−74.72	39.40	3.89	1991–2011	1991, 1995, 2002, 2006,2007, 2008, 2010, 2011	Eurasian	6	1299.26±714.16	4	0
Appoquinimink River	DL	−75.67	39.43	1.00	1989–2009	1989, 1997, 2006, 2009	Eurasian	16	3116.01±1780.68	4	0
Mackay Island NWR*	NC	−75.94	36.51	17.48	1993–2010	1993, 1998, 2005, 2006,2008, 2009, 2010	Eurasian	6	1468.97±389.20	10	1
Georgetown	SC	−79.26	33.37	12.10	1990–2011	1990, 1994, 1999, 2005,2006, 2009, 2011	Eurasian	7	592.79±206.12	12	2
Apalachicola Bay	FL	−84.97	29.72	8.48	1984–2010	1984, 1995, 1999, 2004,2007, 2010	Gulf-Coast	7	2343.99±1008.56	8	2
Mobile Bay	AL	−87.95	30.66	15.68	2006–2011	2003, 2006, 2009, 2011	Gulf-Coast	8	276.99±123.20	3	1
Delta NWR	LA	−89.19	29.13	20.03	1983–2010	1983, 1994, 1998, 2004,2005, 2007, 2009, 2010	Eurasian	7	271.40±128.35	10	5
Big Branch Marsh NWR	LA	−89.82	30.25	1.00	1998–2010	1988, 1998, 2004, 2005,2007, 2009, 2010	Gulf-Coast	2	960.61±354.20	6	1
Intracoastal City	LA	−92.20	29.78	25.00	1998–2010	1994, 1998, 2003,2005, 2010	Gulf-Coast	6	1806.27±820.15	2	2
Rockefeller WR**	LA	−92.83	29.68	25.00	1988–2010	1988, 1994, 1998, 2003,2005, 2008, 2009, 2010	Eurasian	8	1317.67±279.94	7	2
Cameron Prairie NWR	LA	−93.08	29.95	8.62	2003–2010	1998, 2003, 2005,2007, 2008,2009, 2010	Eurasian	16	387.88±98.11	5	2
Sabine NWR	LA	−93.44	29.86	4.01	1998–2010	1994, 1998, 2003,2005, 2007,2009, 2010	Eurasian	5	272.65±87.71	5	2

*NWR = National Wildlife Refuge, **WR = Wildlife Refuge.

## Materials and Methods

### Study Sites and Estimation of Growth Rate

We selected 13 freshwater-to-brackish marshes distributed along the Gulf and Atlantic Coasts of the US that were occupied by one of two non-indigenous haplotypes of *P. australis* that exhibit both aggressive patterns of local spread and range expansion [22], [25]–[27] (Figure 1, Table 1). Because patches of native haplotypes are difficult to distinguish from the background marsh vegetation in historical aerial images (GP Bhattarai, JT Cronin, WJ Allen, LA Meyerson unpublished data), the native haplotypes were excluded from this study. We selected relatively undisturbed open marsh habitats where *P. australis* was at early stage of invasion and had the potential to grow. Eight of our sites were located in protected areas (wildlife refuges, management areas and state parks) but all 13 sites were relatively undisturbed during the study period. All sites along the Atlantic coast and four sites in Louisiana were occupied by introduced Eurasian haplotype. The remaining four sites (one in Florida, one in Alabama, and two in Louisiana) were occupied by a non-native Gulf-Coast haplotype.

*P. australis* patches were identified initially based on morphological characters and, then, confirmed by an analysis of the chloroplast DNA [22]. Marsh sites were ≤25 km from the ocean or gulf and could potentially flood from the storm surge. Most of the sites were tidal but the sites along the Gulf Coast experience smaller tides in comparison to those in the Atlantic Coast. These sites were separated from each other by at least 40 km and none shared the same drainage system.

Within each site, we selected a 1–25 km^2^ area within the interior of the marsh that contained discrete *P. australis* patches (Table 1). Patches within this area were unconstrained by any physical barriers to expansion (e.g., roads, bodies of water, agricultural lands, marsh edges). These dense and usually circular patches of *P. australis* were readily identifiable in aerial images (color, color-infrared and black-and-white images) because of their distinct color and texture against the background marsh vegetation [31], [32].

Twenty to thirty *P. australis* patches were identified in the most recent set of aerial images available for each site, and digitized in ArcMAP 10.1 (ESRI, Redlands, CA). The existence of those patches was verified during field visits to the sites. Patches were then followed backward in time through a series of aerial images to the early 1980s or until they were no longer visible on the images. Only those patches which were present in the oldest set of imagery were considered in this study. The number of focal patches within each site averaged 8.15±1.14 (mean ± SE; range: 2–16, Table 1). Annual growth rate per patch was determined as the proportional change in area per year: *ln* [(final patch area/initial patch area)]/number of years [31]. Clonal growth is expected to be the primary means of *P. australis* patch expansion [33] but we cannot rule out the contribution of sexual reproduction [34]. For each marsh site, an average growth rate was computed from the collection of focal patches.

### Hurricane and Tropical Storm Frequency

We used wind speed as an indicator of the strength of the storm as a disturbance to *P. australis*. Data on other disturbances associated with tropical storms and hurricanes (e.g., storm surge, change in salinity, nutrient levels, deposition of silt and wrack) are mostly unavailable. However, it is likely for coastal marshes that wind speed is correlated with these other variables.

Information about hurricane and tropical storm (tropical and sub-tropical) tracks and maximum wind speeds along those tracks were collected from the International Best Track Archive for Climate Stewardship (IBTrACS, v03r04 WMO) for the North American Basin [35]. Using ArcMAP 10.1, storm tracks passing within a radius of 200 km around each study site during the study period were extracted. The maximum wind speed of each storm in the study site was estimated using the Rankine combined vortex approximation model [36]. First, the minimum distance between the center of the study site and storm track was determined for each storm. Second, because the radius of maximum winds for a hurricane is estimated to be 48 km [37], if the storm passed within this distance of the study-site center, the maximum sustained wind speed was considered the wind speed experienced at the site. For the storms more than 48 km from the site center, maximum sustained wind speed for that site was estimated as
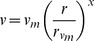
where 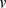 is wind speed at the site, 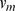 is the maximum wind speed, 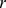 is the distance between the site and hurricane path, 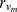 is the radius of maximum winds, and 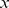 is the scaling parameter [36]. We used 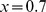 as recommended by Hsu and Babin [38].

All storm events with wind speeds ≥35 knots (64.9 km/hr), the minimum for categorization of a tropical storm based on the Saffir-Simpson hurricane wind scale [39], were included in the analysis. For each site, storms were categorized as either tropical or sub-tropical storms (35–64 knots, or 64.9–118.5 km/hr) or hurricanes (above 64 knots or 118.6 km/hr) based on a popular convention. A total of 79 tropical and sub-tropical storms and 21 hurricanes (average wind speed = 99.47 km/hr, SE = 3.11, range = 65–231.5 km/hr) passed through our sites during the study period. Annual frequencies of tropical storms and hurricanes were determined for each site.

### Climate Data

One of the objectives of this study was to evaluate whether *P. australis* growth rates were influenced more by large-scale storm events than by local climatic conditions. To this end, the following climate data for each site were obtained from the BIOCLIM database [40]: annual mean temperature, isothermality (mean of monthly [maximum temperature – minimum temperature]/annual temperature range), temperature seasonality (standard deviation of weekly mean temperatures), maximum temperature of warmest month, minimum temperature of coldest month, temperature annual range, annual precipitation, precipitation seasonality (standard deviation of weekly mean precipitation estimates expressed as the percentage of mean of those estimates), precipitation of wettest quarter, and precipitation of driest quarter. A principal component analysis was run to reduce the dimensionality of climatic data. The first two principal components, which explained 94.8% and 5.01% variability of the climatic data respectively, were used in our model-selection procedure.

### Model Selection

We examined the effects of latitude, initial patch size, climatic variables (PC1 and PC2), frequency of tropical storms, and frequency of hurricanes on growth rate of *P. australis* patches. Using general linear models in Systat 12 (Systat Inc., Chicago, IL), we developed statistical models using all combinations of latitude (*x_1_*), initial patch size (*x_2_*), PC1 (*x_3_*), PC2 (*x_4_*), frequencies of tropical storms (*x_5_*) and hurricanes (*x_6_*). The best model was selected using corrected Akaike weights [41] (Table S1). The time interval over which *P. australis* growth was measured for each site (*P* = 0.11) and intensity of hurricanes (sum total of hurricane categories [1–5; Saffir-Simpson scale]) (*P* = 0.71) did not have a significant effect on growth rates of patches. Therefore, we did not include them in analysis. Examination of the standardized residuals in our best model showed that one of the data points was an outlier (Intracoastal City, LA). Removal of that point in the analysis improved the fit of the model to the data (*F*_2,9_ = 36.53, *P* = 0.001, *R* ^2^ = 0.89). Because we have no reason to conclude that this data point is spurious, we retained it in our analysis.

## Results/Discussion

**Figure 2:**
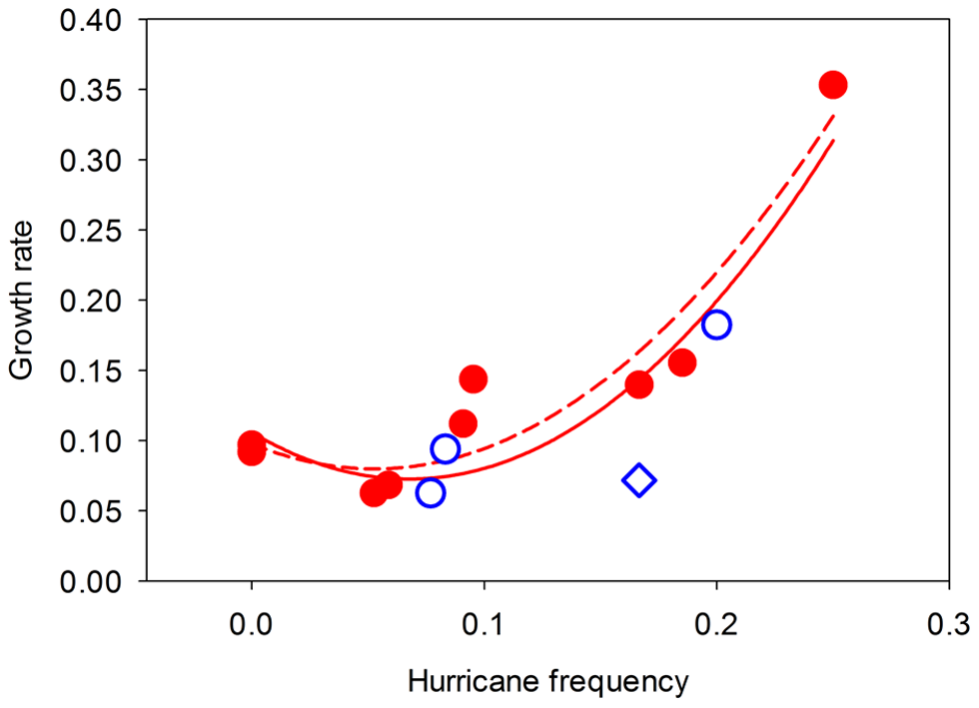
Effect of hurricane frequency on *P. australis* growth. Annual growth rate (proportional change in *ln* area) of *P. australis* patches as a function of hurricane frequency in the coastal marshes of the United States. Filled and open symbols represent sites occupied by Eurasian and Gulf-Coast haplotypes respectively. Solid curve is the best-fit model representing all sites (*F*_2,10_ = 21.66, *P*<0.001, *R^2^* = 0.81). The diamond-shaped symbol was identified as an outlier based on the examination of standardized residuals. The relationship was still significant when it was removed from the analysis (*P*<0.001, *R* ^2^ = 0.89). The dotted curve represents the best-fit model for only the sites occupied by the Eurasian haplotype (*F*_2,6_ = 26.87, *P = *0.001, *R = *0.90).

**Figure 3:**
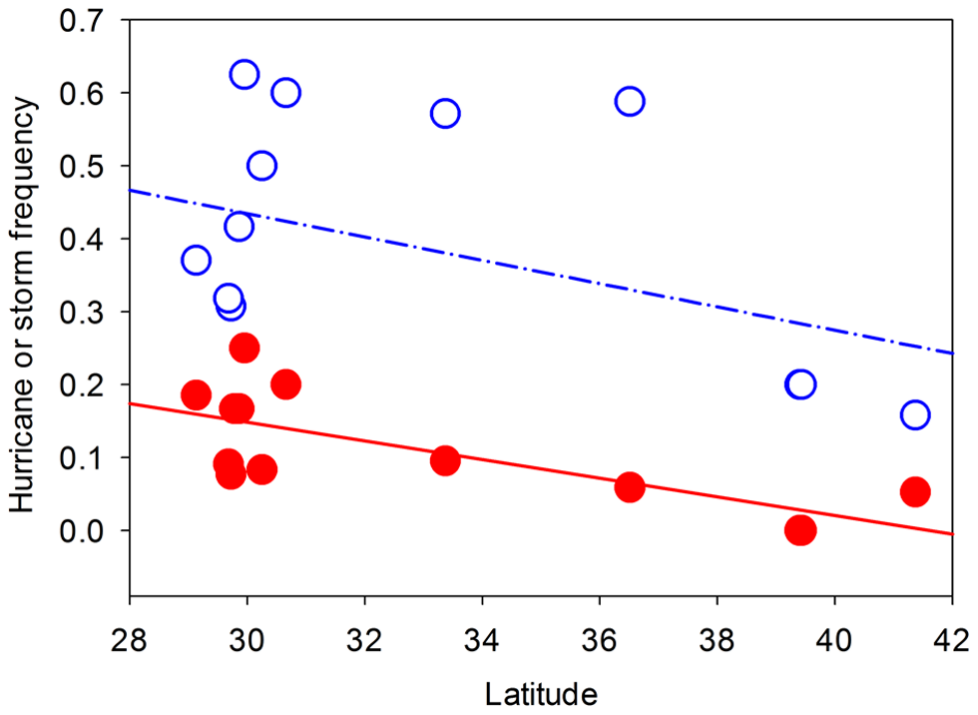
Relationship between the tropical storm and hurricane frequencies (number per year) and latitude. Filled and open symbols represent hurricane and tropical storm frequencies respectively. Lines for each storm category are fit by separate least-squares regression analyses (Tropical storms: *R* ^2^ = 0.16, *P = *0.16; Hurricanes: *R* ^2^ = 0.55, *P = *0.004).

**Figure 4:**
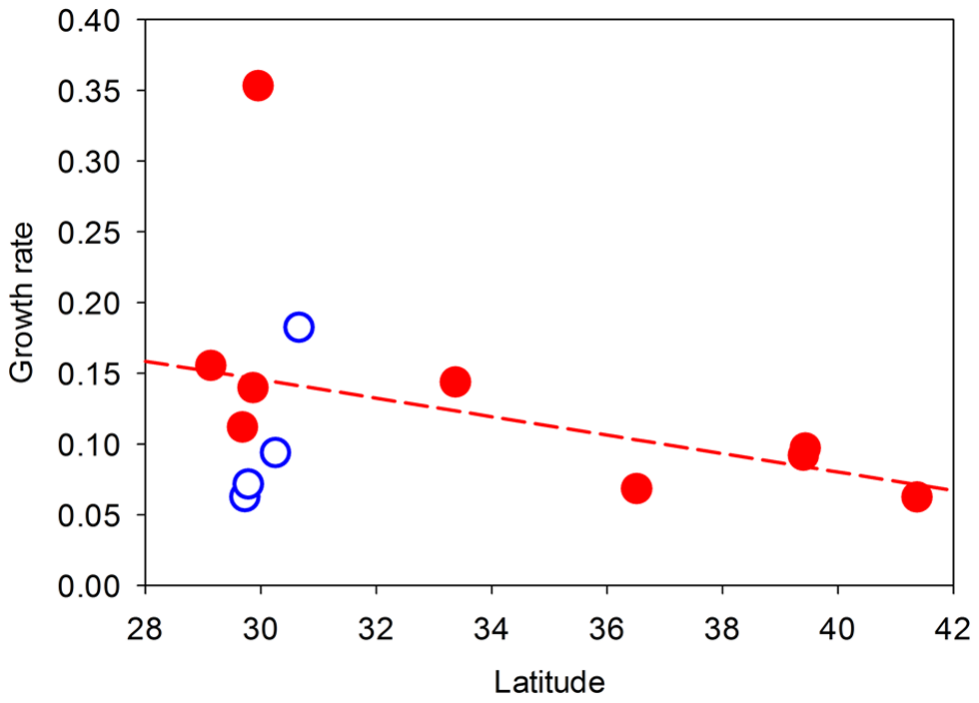
Effect of latitude on *P. australis* growth. Annual growth rate (proportional change in *ln* area) of *P. australis* patches as a function of latitude in the coastal marshes of the United States. Filled and open symbols represent sites occupied by Eurasian and Gulf-Coast haplotypes respectively. Line is fit by least-squares regression (both haplotypes combined; *R* ^2^ = 0.14, *P = *0.20).

Average annual growth rate of *P. australis* patches within a site varied from 6.3% to 35.3% among our sites. The best-fit model for explaining the variation in *P. australis* growth rates among sites included only hurricane frequency (Table S1; *P. australis* growth rate = *a*[hurricane frequency]+*b*[hurricane frequency]^2^+ *k*; Akaike weights = 0.61, Evidence ratio = 4.72, Normalized evidence ratio = 0.83). The growth rates of *P. australis* patches in semi-protected coastal marshes of the US (Eurasian and Gulf-Coast haplotypes combined) increased significantly, but nonlinearly, with hurricane frequency (Figure 2). Eighty-one percent of the variation in *P. australis* growth rate was explained by just this one abiotic factor. Interestingly, the occurrence of lower-intensity storms did not contribute in an appreciable way to the growth of *P. australis* patches (Table S1). Hurricane frequency was greatest in the south and decreased with increasing latitude (*P* = 0.004) but storm frequency was independent of latitude (*P* = 0.16, Figure 3). Despite these latitudinal patterns, latitude was uncorrelated with *P. australis* growth rates (*P* = 0.20, Figure 4). Growth rates of *P. australis* patches were also independent of the climatic variables (Table S1).

Although it would have been desirable to statistically test whether the growth rates of the Eurasian and Gulf-Coast haplotypes responded differently to hurricane frequency, we did not have sufficient replication for the Gulf-Coast haplotype to allow for this comparison. However, we were able to compare the best-fit model for the growth rate of *P. australis* with and without sites representing the Gulf-Coast haplotype. Exclusion of sites with the Gulf-Coast haplotype did not alter the results (*F*_2,6_ = 26.87, *P* = 0.001, *R^2^* = 0.90, Figure 2). Moreover, a comparison of the growth rates of sites occupied by Gulf-Coast haplotype with those of sites occupied by Eurasian haplotype indicates that growth rates were not significantly different (Eurasian: 0.13±0.03 [mean ± SE]; Gulf-Coast: 0.10±0.03 [mean ± SE]; *t*_11_ = 0.69, *P* = 0.50). These results suggest that the Gulf-Coast haplotype is not only spreading rapidly in marshes along the Gulf Coast of the US but also is responding to disturbance events similarly to the well-known Eurasian invader.

The strong positive correlation between hurricane frequency and the patch growth rate of the Eurasian and Gulf-Coast haplotypes reveals the importance of large-scale disturbances on biological invasions. Severe destruction of natural vegetation accompanied with drastic changes in habitat characteristics including hydrology, salinity, and geomorphology [42]–[44] should create room for the spread of an invasive plant [16]. *P. australis* is one of the early species to recover after a major hurricane [42]. An extensive underground system of rhizomes and roots representing over two-thirds of the total biomass of *P. australis* may enable this species to survive catastrophic disturbances and re-sprout much earlier than the native vegetation. In coastal-area marshes, storm surge brought about by hurricanes often results in temporary flooding and elevated salinity [42]. Increased salinity in freshwater and brackish marshes may on its own, or in combination with the damage from winds, inhibit the recovery of native species. In the case of the Eurasian and Gulf-Coast haplotypes of *P. australis,* which have been shown to tolerate mesohaline levels of salinity [45], [46], storm surge may greatly increase their competitive advantage over native species. Alternatively, excessive rainfall during hurricanes which could account up to 40% of total annual precipitation in a site may reduce salinity in hypersaline marshes [43], [47] allowing for establishment and growth of *P. australis* in these environments. Increased concentration of organic matter in the wetland following a hurricane event [42], [43], [48] may also benefit the growth of plant species like *P. australis* that are amongst the first species to recover from a hurricane. The fact that we found no effect of tropical storm frequency on *P. australis* growth rates suggests that these lower wind-speed storms may not sufficiently free up resources, or alter salinity and nutrient levels, to an extent that favors increased growth of *P. australis*. Although we could not estimate the emergence rate of new stands from aerial images (because patches appeared and merged too quickly), it is likely the case that increased hurricane activity also caused an increase in the proliferation of new *P. australis* patches.

The invasion of the Eurasian haplotype of *P. australis* in the Atlantic Coast of the US has been attributed to increased anthropogenic disturbance and nutrient enrichment following coastal development [49]–[54]. Construction of highway networks has also been linked to the spread of the introduced haplotype in Canada [55]. In our study, we specifically selected sites from marshes that were relatively undisturbed by humans to minimize the effects of anthropogenic disturbances on the expansion of patches. Our study provides compelling evidence that large-scale disturbance events can be of overriding importance in the spread of *P. australis* in semi-protected areas in the coastal regions. Hurricane frequency over the past several decades explained over 80% of the variation in the growth rates of *P. australis* patches across the Gulf and Atlantic Coasts of the US. In this case, *P. australis* growth rates were greater along the Gulf and southern Atlantic Coast where hurricanes occurred more frequently. This geographic pattern in growth rates appears to be driven by factors associated with hurricanes, not other climatic or environmental variables associated with latitude because growth rates were unrelated to latitude (Figure 4) and climatic variables in our statistical models (Table S1). However, the contribution of specific components of hurricane disturbance (e.g., storm surge, nutrient fluxes, changes in salinity) on the growth rate of *P. australis* has yet to be evaluated.

A high priority in the future should be a comparison of the growth rates of native and the European exotic haplotypes in response to large-scale disturbance events. Native haplotypes are found in coastal marshes from North Carolina to Canada ([22]; JT Cronin, GP Bhattarai, WJ Allen, LA Meyerson unpublished data) and are present in four of our study sites (North Carolina, Delaware, New Jersey and Connecticut). In general, patches of native haplotypes are rare (in terms of area of coverage) and are thought to be disappearing, in part, due to the invasion of exotic *P. australis* [22], [26]. Because native haplotypes are reported to be less tolerant to disturbances and salinity levels [45], we would expect that they may respond less positively, or even negatively, to hurricane events. With higher resolution color and infrared imagery, hyperspectral imagery, and LIDAR [31], [32], it should be possible in the future to map the growth and spread of native haplotypes over time.

Our study suggests that in semi-protected areas like national wildlife refuges and preserves, where introduced *P. australis* has invaded, the outlook is dire. Left unchecked, nonnative haplotypes grow very rapidly. The end result is likely to resemble areas like the Chesapeake Bay and the New Jersey Meadowlands which are now dominated by *P. australis*. In protected areas where chemical control may not be an option, management of *P. australis* poses a great challenge. The management of this species through biological control does not appear promising, as most herbivores prefer and perform better on the rare native haplotypes ([56]; GP Bhattarai, WJ Allen, LA Meyerson, JT Cronin unpublished data). Biological control using fungal pathogens is under consideration [57] but this approach is likely also to be limited by the need for within-species specificity. Mechanical removal during the early stages of invasion has been employed [58] but those methods are costly, labor intensive, and potentially damaging to the hydrology of the system and neighboring plants [58], [59]. Unfortunately, this may be the only option available to managers of these sensitive lands. In areas where *P. australis* is just beginning to arrive, managers must respond immediately to the threat.

Many of our most notable species invasions have occurred or are occurring at continent-wide scales. To date, studies of these biological invasions have rarely considered the possibility that large-scale phenomena may underlie geographic variation in invader establishment and spread. Recent studies on the effects of global climate change on biological invasions [4], [5] are an important step in the right direction but clearly more attention needs to be paid to other large-scale climatic disturbances (e.g., storms, droughts) and their effects on all types of invasive species, not just plants.

Understanding the role of hurricanes and storms in biological invasions is particularly relevant in the current context of global climate change. Sea surface temperature has been shown to strongly relate to tropical storm and hurricane activity [60] suggesting a recent increase in storm counts and their destructiveness [19], [21]. Although still a very contentious issue, some climatic models predict an increase in the intensity and frequency of storms in the future ([20], [21]; *but see* [61]). This does not bode well for the susceptibility of coastal ecosystems to the future establishment and spread of invasive species.

## Supporting Information

Table S1 **Comparison of models estimating the effects of latitude (** ***x_1_*** **), patch size (** ***x_2_*** **), PC1 (** ***x_3_*** **), PC2 (** ***x_4_*** **), tropical storm frequency (** ***x_5_*** **), hurricane frequency (** ***x_6_*** **) on mean patch growth rate (** ***y*** **).**
(PDF)
