# Supplementary material for: Hurricane Activity and the Large-Scale Pattern of Spread of an Invasive Plant Species
Source: PLoS One. 2014 May 30;9(5):e98478. doi: 10.1371/journal.pone.0098478 (PMC4039472; doi:10.1371/journal.pone.0098478)
Supplement: Table S1 — Comparison of models estimating the effects of latitude ( x1 ), patch size ( x2 ), PC1 ( x3 ), PC2 ( x4 ), tropical storm frequency ( x5 ), hurricane frequency ( x6 ) on mean patch growth rate ( y ). (PDF) [file pone.0098478.s001.pdf]

**Table S1.** Comparison of models estimating the effects of latitude ($x_1$), patch size ($x_2$), PC1 ($x_3$), PC2 ($x_4$), tropical storm frequency ($x_5$), hurricane frequency ($x_6$) on mean patch growth rate ($y$).

| Model | Number of Parameters | AICc | $w_i$(AICc) |
|---|---|---|---|
| $y = k$ | 1 | -25.150 | 0.001 |
| $y = ax_1 + k$ | 2 | -23.665 | 0.000 |
| $y = ax_2 + k$ | 2 | -27.238 | 0.002 |
| $y = ax_3 + k$ | 2 | -22.746 | 0.000 |
| $y = ax_4 + k$ | 2 | -22.550 | 0.000 |
| $y = ax_5 + k$ | 2 | -27.329 | 0.002 |
| $y = ax_6 + k$ | 2 | -31.584 | 0.014 |
| $y = ax_1 + bx_2 + k$ | 3 | -22.915 | 0.000 |
| $y = ax_1 + bx_3 + k$ | 3 | -19.836 | 0.000 |
| $y = ax_1 + bx_4 + k$ | 3 | -20.890 | 0.000 |
| $y = ax_1 + bx_5 + k$ | 3 | -23.428 | 0.000 |
| $y = ax_1 + bx_6 + k$ | 3 | -29.093 | 0.004 |
| $y = ax_2 + bx_3 + k$ | 3 | -22.936 | 0.000 |
| $y = ax_2 + bx_4 + k$ | 3 | -23.754 | 0.000 |
| $y = ax_2 + bx_5 + k$ | 3 | -24.216 | 0.000 |
| $y = ax_2 + bx_6 + k$ | 3 | -27.825 | 0.002 |
| $y = ax_3 + bx_5 + k$ | 3 | -23.108 | 0.000 |
| $y = ax_3 + bx_6 + k$ | 3 | -33.057 | 0.029 |
| $y = ax_4 + bx_5 + k$ | 3 | -23.902 | 0.000 |
| $y = ax_4 + bx_6 + k$ | 3 | -27.924 | 0.002 |
| $y = ax_3 + bx_4 + k$ | 3 | -19.355 | 0.000 |
| $y = ax_3 + bx_4 + cx_5 + k$ | 4 | -18.471 | 0.000 |
| $y = ax_3 + bx_4 + cx_6 + k$ | 4 | -27.831 | 0.002 |
| $y = ax_5 + bx_6 + k$ | 3 | -29.307 | 0.005 |
| $y = ax_1 + bx_2 + cx_3 + k$ | 4 | -17.385 | 0.000 |
| $y = ax_1 + bx_2 + cx_4 + k$ | 4 | -18.208 | 0.000 |
| $y = ax_2 + bx_3 + cx_4 + k$ | 4 | -18.200 | 0.000 |
| $y = ax_1 + bx_2 + cx_5 + k$ | 4 | -18.645 | 0.000 |
| $y = ax_1 + bx_2 + cx_6 + k$ | 4 | -25.577 | 0.001 |
| $y = ax_1 + bx_5 + cx_6 + k$ | 4 | -26.258 | 0.001 |
| $y = ax_3 + bx_5 + cx_6 + k$ | 4 | -30.374 | 0.008 |
| $y = ax_2 + bx_3 + cx_5 + k$ | 4 | -18.666 | 0.000 |
| $y = ax_2 + bx_4 + cx_5 + k$ | 4 | -19.536 | 0.000 |
| $y = ax_2 + bx_3 + cx_6 + k$ | 4 | -28.818 | 0.004 |
| $y = ax_2 + bx_4 + cx_6 + k$ | 4 | -22.967 | 0.000 |
| $y = ax_2 + bx_5 + cx_6 + k$ | 4 | -23.739 | 0.000 |
| $y = ax_4 + bx_5 + cx_6 + k$ | 4 | -24.507 | 0.000 |
| $y = ax_1 + bx_2 + cx_3 + dx_5 + k$ | 5 | -11.388 | 0.000 |
| $y = ax_1 + bx_2 + cx_3 + dx_6 + k$ | 5 | -21.806 | 0.000 |

| | | | |
|---|---|---|---|
| $y = ax_1 + bx_2 + cx_4 + dx_5 + k$ | 5 | -12.200 | 0.000 |
| $y = ax_1 + bx_2 + cx_4 + dx_6 + k$ | 5 | -18.185 | 0.000 |
| $y = ax_2 + bx_3 + cx_4 + dx_5 + k$ | 5 | -12.118 | 0.000 |
| $y = ax_2 + bx_3 + cx_4 + dx_6 + k$ | 5 | -21.766 | 0.000 |
| $y = ax_3 + bx_4 + cx_5 + dx_6 + k$ | 5 | -23.377 | 0.000 |
| $y = ax_5 + bx_5^2 + k$ | 3 | -23.903 | 0.000 |
| $y = ax_1 + bx_5 + cx_5^2 + k$ | 4 | -20.569 | 0.000 |
| $y = ax_2 + bx_5 + cx_5^2 + k$ | 4 | -21.663 | 0.000 |
| $y = ax_3 + bx_5 + cx_5^2 + k$ | 4 | -18.863 | 0.000 |
| $y = ax_4 + bx_5 + cx_5^2 + k$ | 4 | -18.801 | 0.000 |
| $y = ax_3 + bx_4 + cx_5 + dx_5^2 + k$ | 5 | -11.828 | 0.000 |
| **$y = ax_6 + bx_6^2 + k$*** | **3** | **-39.113** | **0.608**** |
| $y = ax_1 + bx_6 + cx_6^2 + k$ | 4 | -33.797 | 0.043 |
| $y = ax_2 + bx_6 + cx_6^2 + k$ | 4 | -36.009 | 0.129 |
| $y = ax_3 + bx_6 + cx_6^2 + k$ | 4 | -33.654 | 0.040 |
| $y = ax_4 + bx_6 + cx_6^2 + k$ | 4 | -33.543 | 0.038 |
| $y = ax_3 + bx_4 + cx_6 + dx_6^2 + k$ | 5 | -26.229 | 0.001 |
| $y = ax_5 + bx_5^2 + cx_6 + dx_6^2 + k$ | 5 | -34.173 | 0.051 |
| $y = ax_1 + bx_2 + cx_5 + dx_5^2 + k$ | 5 | -14.914 | 0.000 |
| $y = ax_1 + bx_2 + cx_6 + dx_6^2 + k$ | 5 | -28.585 | 0.003 |
| $y = ax_2 + bx_3 + cx_5 + dx_5^2 + k$ | 5 | -14.243 | 0.000 |
| $y = ax_2 + bx_3 + cx_6 + dx_6^2 + k$ | 5 | -28.748 | 0.003 |
| $y = ax_2 + bx_4 + cx_5 + dx_5^2 + k$ | 5 | -14.316 | 0.000 |
| $y = ax_2 + bx_4 + cx_6 + dx_6^2 + k$ | 5 | -28.580 | 0.003 |
| $y = ax_2 + bx_3 + cx_5 + dx_6 + k$ | 5 | -23.015 | 0.000 |
| $y = ax_2 + bx_4 + cx_5 + dx_6 + k$ | 5 | -17.081 | 0.000 |
| $y = ax_1 + bx_2 + cx_3 + dx_4 + ex_5 + k$ | 6 | -2.920 | 0.000 |
| $y = ax_1 + bx_2 + cx_3 + dx_4 + ex_6 + k$ | 6 | -12.722 | 0.000 |
| $y = ax_1 + bx_2 + cx_3 + dx_5 + ex_6 + k$ | 6 | -13.533 | 0.000 |
| $y = ax_1 + bx_2 + cx_4 + dx_5 + ex_6 + k$ | 6 | -8.889 | 0.000 |
| $y = ax_2 + bx_3 + cx_4 + dx_5 + ex_6 + k$ | 6 | -13.046 | 0.000 |
| $y = ax_1 + bx_2 + cx_3 + dx_5 + ex_5^2 + k$ | 6 | -7.064 | 0.000 |
| $y = ax_1 + bx_2 + cx_3 + dx_6 + ex_6^2 + k$ | 6 | -18.530 | 0.000 |
| $y = ax_1 + bx_2 + cx_4 + dx_5 + ex_5^2 + k$ | 6 | -4.728 | 0.000 |
| $y = ax_1 + bx_2 + cx_4 + dx_6 + ex_6^2 + k$ | 6 | -18.185 | 0.000 |
| $y = ax_2 + bx_3 + cx_4 + dx_5 + ex_5^2 + k$ | 6 | -3.951 | 0.000 |
| $y = ax_2 + bx_3 + cx_4 + dx_6 + ex_6^2 + k$ | 6 | -18.350 | 0.000 |
| $y = ax_2 + bx_5 + cx_5^2 + dx_6 + ex_6^2 + k$ | 6 | -23.886 | 0.000 |
| $y = ax_1 + bx_5 + cx_5^2 + dx_6 + ex_6^2 + k$ | 6 | -23.790 | 0.000 |
| $y = ax_3 + bx_5 + cx_5^2 + dx_6 + ex_6^2 + k$ | 6 | -23.914 | 0.000 |
| $y = ax_4 + bx_5 + cx_5^2 + dx_6 + ex_6^2 + k$ | 6 | -23.819 | 0.000 |
| $y = ax_3 + bx_4 + cx_5 + dx_5^2 + ex_6 + fx_6^2 + k$ | 7 | -8.386 | 0.000 |
| $y = ax_1 + bx_2 + cx_3 + dx_4 + ex_5 + fx_5^2 + k$ | 7 | 6.826 | 0.000 |

| | | | |
|---|---|---|---|
| $y = ax_1 + bx_2 + cx_3 + dx_4 + ex_6 + fx_6^2 + k$ | 7 | -3.055 | 0.000 |
| $y = ax_1 + bx_2 + cx_3 + dx_4 + ex_5 + fx_6 + k$ | 7 | -0.061 | 0.000 |
| $y = ax_1 + bx_2 + cx_3 + dx_4 + ex_5 + fx_5^2 + gx_6 + hx_6^2 + k$ | 9 | 69.006 | 0.000 |

* Best model based on AIC weights.

** Evidence ratio of the best model = 4.72, normalized evidence ratio = 0.82.
